# Supplementary material for: Professional quality of life in animal research personnel is linked to retention & job satisfaction: A mixed-methods cross-sectional survey on compassion fatigue in the USA
Source: PLoS One. 2024 Apr 16;19(4):e0298744. doi: 10.1371/journal.pone.0298744 (PMC11020707; doi:10.1371/journal.pone.0298744)
Supplement: Attachment — Submitted filename: Response to Reviewers.docx [file pone.0298744.s003.docx]

November 15, 2023

Dear Dr. Rosemary Frey

Thank you for inviting us to submit a revised version of our manuscript. We appreciate the detailed and helpful feedback that both reviewers provided. We believe their contributions have strengthened our manuscript. We have completed all revisions and address each comment individually below. The reviewer's comments are bolded and our responses are non-bolded.

We would also like to add the following requested statement to the Competing Interests section of our manuscript:

*“This does not alter our adherence to PLOS ONE policies on sharing data and materials.”*

We have also added the following three references to our reference list, based on helpful suggestions from Reviewer #2:

*32. Murray J, Bauer C, Vilminot N, Turner PV. Strengthening workplace well-being in research animal facilities. Frontiers in Veterinary Science. 2020;7. doi:10.3389/fvets.2020.573106*

*33. Tsang B, Gerlai R. Researchers, animal support and regulatory staff: symbiosis or antagonism? Laboratory Animal Research. 2022;38: 19. doi:10.1186/s42826-022-00129-0*

*34. Paull GC, Lee CJ, Tyler CR. Beyond compliance: harmonising research and husbandry practices to improve experimental reproducibility using fish models. Biological Reviews. 2023. doi: 10.1111/brv.13020*

We look forward to receiving your response and decision. Thank you for inviting our revision.

Sincerely,

Megan LaFollette, MS, PhD and Co-Authors

**Journal requirements:**

**1.** **When submitting your revision, we need you to address these additional requirements. Please ensure that your manuscript meets PLOS ONE's style requirements, including those for file naming. The PLOS ONE style templates can be found at**

[**https://journals.plos.org/plosone/s/file?id=wjVg/PLOSOne_formatting_sample_main_body.pdf**](https://journals.plos.org/plosone/s/file?id=wjVg/PLOSOne_formatting_sample_main_body.pdf) **and**

[**https://journals.plos.org/plosone/s/file?id=ba62/PLOSOne_formatting_sample_title_authors_affiliations.pdf**](https://journals.plos.org/plosone/s/file?id=ba62/PLOSOne_formatting_sample_title_authors_affiliations.pdf)

We have revised the manuscript to ensure it meets all of PLOS ONE’s style requirements and have fixed all naming conventions.

**2. Thank you for stating the following in the Competing Interests section:**

**“All authors are employed by the institution noted in their affiliation except for Lauren Young who is a student. All authors are also members of the 3Rs Collaborative’s Compassion Fatigue Initiative. Elizabeth Nunamaker and Sally Thompson-Iritani also sat on the board of directors and leadership team of the 3Rs Collaborative during publication.”**

**Please confirm that this does not alter your adherence to all PLOS ONE policies on sharing data and materials, by including the following statement: "This does not alter our adherence to  PLOS ONE policies on sharing data and materials.” (as detailed online in our guide for authors http://journals.plos.org/plosone/s/competing-interests).  If there are restrictions on sharing of data and/or materials, please state these. Please note that we cannot proceed with consideration of your article until this information has been declared.**

**Please include your updated Competing Interests statement in your cover letter; we will change the online submission form on your behalf.**

We have added the requested line to our Competing Interests statement in the above cover letter. Thank you for updating this on our behalf. We have also added this to our manuscript file.

**3. We note that you have stated that you will provide repository information for your data at acceptance. Should your manuscript be accepted for publication, we will hold it until you provide the relevant accession numbers or DOIs necessary to access your data. If you wish to make changes to your Data Availability statement, please describe these changes in your cover letter and we will update your Data Availability statement to reflect the information you provide.**

This is still correct. We will provide repository information for our data, and there are no changes to our Data Availability statement.

**4. Please remove your figures from within your manuscript file, leaving only the individual TIFF/EPS image files, uploaded separately. These will be automatically included in the reviewers’ PDF.**

We have ensured the figures are removed from within the manuscript, leaving only the image files that were separately uploaded.

**5. Please include captions for your Supporting Information files at the end of your manuscript, and update any in-text citations to match accordingly. Please see our Supporting Information guidelines for more information:** [**http://journals.plos.org/plosone/s/supporting-information**](http://journals.plos.org/plosone/s/supporting-information)**.**

We have added captions for the Supporting Information files to submit along with our manuscript.

**6. Please review your reference list to ensure that it is complete and correct. If you have cited papers that have been retracted, please include the rationale for doing so in the manuscript text, or remove these references and replace them with relevant current references. Any changes to the reference list should be mentioned in the rebuttal letter that accompanies your revised manuscript. If you need to cite a retracted article, indicate the article’s retracted status in the References list and also include a citation and full reference for the retraction notice.**

We have added three new references to our reference list based on suggestions from Reviewer #2.

**Reviewer #1:**

**Dear Authors,**

**Congratulations on your work. It is an interesting manuscript that continues with your line of work and that contributes new data to a field that has more consideration.**

Thank you to Reviewer 1 for these remarks.

1. **Regarding statistics, I would propose to calculate the size effect, and make correlations to determine the influence of each variable studied.**

We have now calculated effect sizes and added these to the Results section of our manuscript at lines 320-325. We briefly mention this in the discussion as well.

2. **There is a typo in table 1, "veterinary technician" 910 instead of 10.**

This is a great catch thank you. This correction to Table 1 has been made.

**Reviewer #2:**

1. **The manuscript by Young et al uses a mixed methods cross sectional survey to explore the quality of life of animal research personnel. Of particular interest is the finding that compassion fatigue is impacted most by institutional culture. On this point, it could be valuable for the authors to divide the respondents by level of seniority of the employee on the institutional culture metric.**

This is a great suggestion. Based on our sample size per role, we only believed it was appropriate to further investigate our themes in animal caretakers and researchers. We do not have large enough sample sizes for veterinarians (n=10), managers (n=18) and the ‘other’ category (n=15), to even make explorative conclusions.

However, we did have a respectable sample size for animal caretakers and researchers. We divided them by the percentage of respondents in each role that mentioned each theme. We have added this to the Results at line 503-510 and have also added a new Figure 3 to display these findings. We have mentioned this in the discussion as well lines 608-612.

2. **As mentioned by the authors, this study cannot account for those who have left their role. However, the inclusion of such data (for example, the turnover rate) of specific job categories, could be insightful. Whether their current institution is their 1st or 5th, and the reason for their departure from previous roles could also provide good info. From my experience, there is often a very high rate of departure and hiring in animal care roles.**

Thank you for this suggestion. We are currently in the midst of updating our survey for another year of data collection, and this is something we will consider adding to our updated survey, to gather data on turnover rates for our longitudinal study.

3. **Line 274 - Table 1 under veterinary technician typo? I believe the authors meant to put n of 10, not 910.**

This is a great catch thank you. This correction to Table 1 has been made.

4. **Papers of potential interest to the authors for this study.**

**https://www.nature.com/articles/s41684-021-00852-6**

**https://labanimres.biomedcentral.com/articles/10.1186/s42826-022-00129-0**

**https://www.frontiersin.org/articles/10.3389/fvets.2020.573106/full**

**https://onlinelibrary.wiley.com/doi/full/10.1111/brv.13020**

Thank you for these references. We have read these publications and have included reference to three of them in our Discussion (lines 561-563 and lines 585-586) to further put our results in the context of the broader literature on compassion fatigue and a Culture of Care.
