# Supplementary material for: Professional quality of life in animal research personnel is linked to retention & job satisfaction: A mixed-methods cross-sectional survey on compassion fatigue in the USA
Source: PLoS One. 2024 Apr 16;19(4):e0298744. doi: 10.1371/journal.pone.0298744 (PMC11020707; doi:10.1371/journal.pone.0298744)
Supplement: Attachment — Submitted filename: Response to Reviewers.docx [file pone.0298744.s004.docx]

January 17, 2024

Dear Dr. Rosemary Frey

Thank you again for inviting us to submit a revised version of our manuscript and allowing us to make these additional revisions. We appreciate the additional feedback that the reviewers have provided. We have completed all revisions and address each comment individually below. The reviewer's comments are bolded, and our responses are non-bolded.

We have also added the following two references to our reference list, based on helpful suggestions from Reviewer #1:

1. *Rumpel, S., Kempen, R., Merle, R., & Thoene-Reineke, C. (2023). Psychological stress and strain in laboratory animal professionals – a systematic review. Laboratory Animals, 57(4), 396–411.* [*https://doi.org/10.1177/00236772221129111*](https://doi.org/10.1177/00236772221129111)
2. *Chang, F. T., & Hard, L. A. (2002). Human-Animal Bonds in the Laboratory: How Animal Behavior Affects the Perspective of Caregivers. ILAR Journal, 43(1), 10–18. https://doi.org/10.1093/ilar.43.1.10*
3. *Friese, C., & Latimer, J. (2019). Entanglements in Health and Well-being: Working with Model Organisms in Biomedicine and Bioscience. Medical Anthropology Quarterly, 33(1), 120–137. https://doi.org/10.1111/maq.12489*
4. *Engel, R. M., Silver, C. C., Veeder, C. L., & Banks, R. E. (2020). Cognitive Dissonance in Laboratory Animal Medicine and Implications for Animal Welfare. Journal of the American Association for Laboratory Animal Science: JAALAS, 59(2), 132–138. https://doi.org/10.30802/AALAS-JAALAS-19-000073*
5. *Goñi-Balentziaga, O., & Azkona, G. (2023). Perceived professional quality of life and mental well-being among animal facility personnel in Spain. Laboratory Animals, 00236772231187177.* [*https://doi.org/10.1177/00236772231187177*](https://doi.org/10.1177/00236772231187177)

We look forward to receiving your response and decision. Thank you for inviting our revision.

Sincerely,

Megan LaFollette, MS, PhD and Co-Authors

**Journal Requirements:**

**Please review your reference list to ensure that it is complete and correct. If you have cited papers that have been retracted, please include the rationale for doing so in the manuscript text, or remove these references and replace them with relevant current references. Any changes to the reference list should be mentioned in the rebuttal letter that accompanies your revised manuscript. If you need to cite a retracted article, indicate the article’s retracted status in the References list and also include a citation and full reference for the retraction notice.**

We have added two additional references per Reviewer #1’s request. We have noted the additional citations in the revised manuscript and in the cover letter above.

**Additional Editor Comments:**

**Please include the two articles requested the reviewer. 1**

We have added the two additional articles per Reviewer #1’s request.

**Reviewers' comments:**

**Review Comments to the Author**

**Reviewer #1: Dear Authors,**

**Thank you for indicating the size effects. During this time I have noticed that two articles have been published in Laboratory Animals that are not mentioned in the bibliography and should be included. The first is a systematic review "Psychological stress and strain in laboratory animal professionals - a systematic review" (10.1177/00236772221129111) and the second is an article also looking at professional quality of life and mental health "Perceived professional quality of life and mental well-being among animal facility personnel in Spain" (10.1177/00236772231187177).**

**Best regards,**

Thank you to Reviewer #1 for this suggestion to add these articles. These are very important findings to mention in comparison to our findings. We have integrated the first article into our Introduction at lines 77-90, as well as three articles that this systematic review references, and we have incorporated its findings into our Discussion at many different points throughout. We have also incorporated the second article into our Discussion at 579-580 and 606. These two articles have also been added to our Reference List, along with three additional articles that Rumpel et al. 2023 reference whose findings we found to be relevant.

**Reviewer #2: Congratulations on the work. This area of study is often overlooked, and I am positive both scientists and animal welfare personnel will benefit from the results of this study to improve their current working policies. Looking forward to reading the follow up studies in the future.**

Thank you to Reviewer #2 for the positive feedback on this work. We also look forward to continuing this work and conducting follow up surveys to build on our findings.
